# The Prediction of Survival Outcome and Prognosis Factor in Association with Comorbidity Status in Patients with Colorectal Cancer: A Research-Based Study

**DOI:** 10.3390/healthcare10091693

**Published:** 2022-09-05

**Authors:** Hafeez Afolabi, Salzihan Md Salleh, Zaidi Zakaria, Ewe Seng Ch’ng, Siti Norasikin Mohd Nafi, Ahmad Aizat Bin Abdul Aziz, Sameer Badri Al-Mhanna, Yusuf Wada, Abdulwali Sabo Abdulrahman

**Affiliations:** 1 Department of General Surgery, School of Medical Sciences, Hospital Universiti Sains Malaysia, Universiti Sains Malaysia USM, Kubang Kerian 16150, Kelantan, Malaysia; 2 Department of Pathology, School of Medical Sciences, Hospital Universiti Sains Malaysia, Universiti Sains Malaysia USM, Kubang Kerian 16150, Kelantan, Malaysia; 3 Advanced Medical and Dental Institute, Universiti Sains Malaysia USM, Kepala Batas 13200, Penang, Malaysia; 4 Department of Pathology, School of Medical Sciences, Universiti Sains Malaysia USM, Kubang Kerian 16150, Kelantan, Malaysia; 5 Department of Human Genome Centre, School of Medical Sciences, Health Campus, Universiti Sains Malaysia USM, Kubang Kerian 16150, Kelantan, Malaysia; 6 Department of Physiology and Exercise, School of Medical Sciences, Health Campus, Universiti Sains Malaysia USM, Kubang Kerian 16150, Kelantan, Malaysia; 7 Department of Medical Microbiology and Parasitology, School of Medical Sciences, Health Campus, Universiti Sains Malaysia USM, Kubang Kerian 16150, Kelantan, Malaysia; 8 Department of Biostatistics and Research Methodology, School of Medical Sciences, Health Campus, Universiti Sains Malaysia USM, Kubang Kerian 16150, Kelantan, Malaysia

**Keywords:** colorectal cancer, survival factors, prognosis factors

## Abstract

Colorectal carcinoma (CRC) is rising exponentially in Asia, representing 11% of cancer worldwide. This study analysed the influence of CRC on patients’ life expectancy (survival and prognosis factors) via clinicopathology data and comorbidity status of CRC patients. **Methodology:** A retrospective study performed in HUSM using clinical data from the Surgery unit from 2015 to 2020. The demographic and pertinent clinical data were retrieved for preliminary analyses (data cleansing and exploration). Demographics and pathological characteristics were illustrated using descriptive analysis; 5-year survival rates were calculated using Kaplan–Meier methods; potential prognostic variables were analysed using simple and multivariate logistic regression analysis conducted via the Cox proportional hazards model, while the Charlson Comorbidity Scale was used to categorize patients’ disease status. **Results:** Of a total of 114 CRC patients, two-thirds (89.5%) were from Malay tribes, while Indian and Chinese had 5.3% each. The 50–69.9 years were the most affected group (45.6%). Overall, 40.4% were smokers (majorly male (95.7%)), 14.0% ex-smokers, and 45.6% non-smokers (*p*-value = 0.001). The Kaplan–Meier overall 5-year median survival time was 62.5%. From the outcomes, patients who were male and >70 years had metastasis present, who presented with per rectal bleeding and were classified as Duke C; and who has tumour in the rectum had the lowest survival rate. Regarding the prognosis factors investigated, “Gender” (adjusted hazard ratio (HR): 2.62; 95% CI: 1.56–7.81, *p*-value = 0.040), “Presence of metastases” (HR: 3.76; 95% CI: 1.89–7.32, *p*-value = 0.010), “Metastasis site: Liver” (HR: 5.04; 95% CI: 1.71–19.05, *p*-value = 0.039), “Lymphovascular permeation” (HR: 2.94; 95% CI: 1.99–5.92, *p*-value = 0.021), and “CEA-level” (HR: 2.43; 95% CI: 1.49–5.80, *p*-value = 0.001) remained significant in the final model for multiple Cox proportional hazard regression analyses. There was a significant mean association between tumour grades and the patient’s comorbidity status. **Conclusions:** Histopathological factors (gender, metastases presence, site of metastases, CEA level, and lymphovascular permeation) showed the best prognosis-predicting factors in CRC.

## 1. Introduction

Colorectal carcinoma (CRC) incidence is rising exponentially in Asia [1], almost similar in pace to the rate seen in the West, especially in countries such as the United States, United Kingdom, Germany, and France [2]. CRC represents 11% of all cancer worldwide [3]; it is the third most prevalent cancer and the third most common cause of cancer-related deaths worldwide, with approximately 700,000 deaths per year globally [4], with an estimated 1.93 million new CRC cases diagnosed and 0.94 million CRC-related caused of deaths in 2020 worldwide. The global new CRC cases are predicted to reach 3.2 million in 2040 (Xi and Xu, 2021). According to the Malaysian National Cancer Registry Report 2007–2011, CRC is the third most frequent cancer among men and second among women. Approximately 25% of patients have distant metastases at the time of diagnosis, and the most common site of metastasis, which develops in 50% of patients with CRC, is the liver. The poor prognosis of metastatic CRC has been the driving force for the ongoing efforts to identify prognosticating factors that can predict survival outcomes and eventually improve patient conditions. CRC prevalence varies regionally, estimated at 30% in the US, 32.1% in the UK, 38.9% in Japan, and 36.9% in Australia [3]. Globally, the CRC burden will rise by 60% to more than 2.2 million new cases and 1.1 million mortality by 2030 [3]

**Figure 1:**
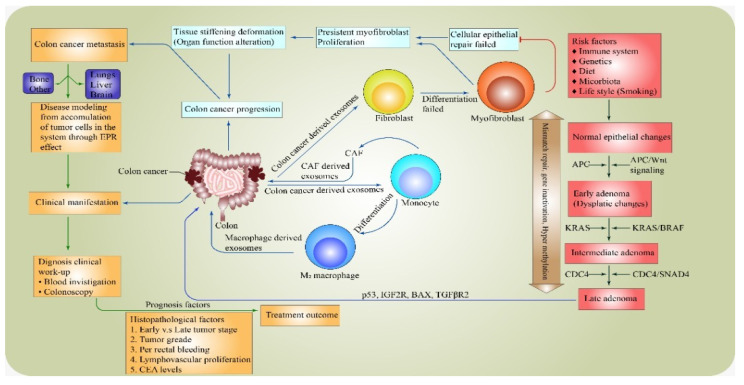
Mechanism and prognosis factors influence the pathogenesis of colorectal cancer.

Colorectal tumorigenesis is a multistep process that involves the accumulation of multiple, successive genetic alterations, including chromosomal abnormalities, gene mutations, and epigenetic changes, which transform the normal colonic epithelium into colorectal carcinoma [5]. Several gene mutations and microsatellite instability are the major culprits in the development and progress of cancerous cells, such as in CRC. This latter statement encourages the transformation of the normal epithelial cells into dysplastic forms that eventually promote cancerous growth (Figure 1). A key to a successful or better prognosis is the early identification of the prognosis factors that, in most cases, predict or influence the treatment outcome, such as when patients present in the early stages of the disease than at the late stage, where the management becomes palliative as metastases have already begun or occurred.

Several blueprints have been put forward as related variables associated with survival post-cancer illness. One effective technique to determine how well a treatment works in a clinical trial is to measure the overall survival status, which is the amount of time that the individuals diagnosed with an illness, such as colorectal cancer, have been alive since the date of diagnosis or the commencement of therapy [6]. The Duke’s stage or TNM staging, number of lymph nodes involved, distant metastases, pre and postoperative carcinoembryonic antigen (CEA) level, tumour grade and location are regarded as prognostic markers with utmost prognostic importance that predict the survival of individuals with colorectal cancer [7,8]. In colorectal cancer, the best prognosis estimate is based on the anatomic extent of illness as established by the pathological inspection of the resected specimen [9]. However, precise prognostic factor identification for colorectal cancer CRC still poses a challenge; thus, further research is required to understand the predictive relevance of clinicopathological factors in CRC, particularly for Malaysian records.

Despite several efforts to diagnose cancer at an early stage, the general long-term result of patients who have been curatively resected has failed to show significant change over the previous decade, with a 5-year survival rate of around 60% [10]. More than half of colorectal adenocarcinomas are detected only when cancer has spread to other body parts, i.e., late stage of the disease [11,12,13]. In today’s world, we are approaching an era of personalised and precision medicine where cancer diagnosis and treatment will be tailored to each patient depending on their unique genetic signatures to accord potential personalised therapies based on molecular-specific subtypes geared towards ensuring a significant survival status. Hence, through this study, the researchers aimed to determine the pattern of survival outcomes and the comorbidity status influence on prognostic factors of colorectal cancer patients in Universiti Sains Malaysia (HUSM).

## 2. Methodology

A retrospective study was performed in HUSM using data from the clinicopathological unit of the General Surgery department. The clinical records of patients diagnosed with colorectal cancer were retrieved from their case notes and from the faculty medical database from 2010 to 2015. The selected individuals were all patients with cases of colorectal cancer that were diagnosed and treated in the hospital. Still, the eligible source population were those with confirmed CRC and that have available clinical records for the treatments received in the hospital.

Patients’ inclusion criteria include: 1. patients with clinical records and histopathologically confirmed CRC cases; 2. who received treatment at least once in HUSM between 2010 and 2015; and 3. have available medical records detailing medical biodata information’s and the treatment carried out while on admission and as an outpatient. The exclusion criteria include: 1. those patients with no or incomplete clinical records of less than 30%; 2. those patients with loss to follow-up records; and 3. all non-colorectal cancer cases.

The sample size was estimated for the demographic and histopathological characteristics of the patients. We used the G*Power sample size calculator software version 3.0.1, the effect size of 0.8 (large effect), power of 80% (type 11 error rate), and probability error of 5% (type 1 error). The calculated sample size was 96. After adding a 10% non-response rate, the adjusted sample size was 114. The median survival period for the patients receiving standard care and the ratio of control to experimental patients was extrapolated from the literature. The investigator and chief surgeon determined the detectable hazard ratio for the 120 months recruitment duration.

The clinical data for the selected patients were retrieved for demographic, medical data (i.e., gender, age, ethnicity, occupation, and habitual lifestyle), and pertinent clinical presentations. The patient’s age was defined as age in years as recorded on the first day of diagnosis at the time of consultation. The patients’ ethnicity was categorised into three groups: the Malay, Chinese, and Indian races. A three-subclass definition was provided to habitual lifestyle (smoking habit) irrespective of quantity taking: “smokers group are patient actively smoking”, “non-smokers group are patients with no smoking record”, and “ex-smokers group are patients with smoking cessation records before diagnosis”.

The study’s clinical focus parameters include gender, tumour sites, tumour stages, tumour metastases, bleeding per rectum, tumour grading, CEA level, and lymphovascular permeation. Tumour site refers to the tumour location at diagnosis via colonoscopy by the surgeon, which was classified into three categories, namely: the left colon (tumour arises from the descending colon, sigmoid colon, and distal one-third of the transverse colon), right colon (tumours arise from ascending colon, and proximal two-thirds of the transverse colon), and the rectum. The division of tumours into stages was depicted using Duke’s stage, divided into four groups (Dukes A, Dukes B, Dukes C, and Dukes D), with Duke A and B representing “early stages” and Duke C and D representing “late stages”. The presence or absence of metastases was illustrated regardless of the time of commencement, and this was defined as the spreading of the tumour to sites ordered from the primary site (colorectal region). The identification of specific sites of metastases in this study was majorly to the Liver. The occurrence of per rectal bleeding was assessed based on the initial symptoms reported by the patients as mentioned or recorded in the medical records, irrespective of the duration. Preoperative carcinoembryonic antigen CEA level assessment was illustrated via two categories of the CEA level, ≤5 ng/mL and >5 ng/mL, with a CEA value of >5 ng/mL being regarded as abnormal. Regarding the comorbidity status, the assessment was categorized into three groups based on the number of chronic diseases reported in the medical note: “Single”, “Two or more”, and “None”. The study was carried out in line with the code and conduct of the institution’s ethics committee; ethical approval was obtained from the Universiti Sains Malaysia (USM) Human Research Ethics Committee USM/JEPeM/21010076.

Charlson Comorbidity Index (CCI) and Score CCIS. The CCI is deemed the most utilized medical comorbidity-determining scale [14] invented for the categorization of chronic comorbidities for the first time in 1987 [15], and the publication has gained almost 5500 citations [16]. The arising question is why is CCI essential? By designating the CCIS to comorbidities, medical professionals can decide how thorough or assertively to treat any specific illness, and this is because the patient is often ignorant of the seriousness of their health conditions. CCI comprises 17 lists of prominent chronic diseases (Charlson comorbidities) and their assigned weight index (Table 1). CCI is a means for obtaining follow-up on patient’s health status to foretell the health-functional outcome of the patient.

By assigning a certain weighted number to a specific disease, the CCI-Calculator software was able to calculate the CCI Score (CCIS) for the comorbid status of the patients, which, in turn, can be used to categorize the comorbidity status of the patient into Mild: CCI score of 1–2, Moderate: CCI score of 3–4, Severe: CCI score of ≥5, and None: CCI score of zero, shown in Table 2 below. The obtained weighted CCIS represents the patient’s cumulated comorbidity status that is weighted from a scale of (0, 1, 2, 3, 4, and 5). A higher CCIS indicates a more severe condition, meaning the more points awarded, the more likely the predicted negative outcome.

Firstly, the preliminary analysis was performed for data cleansing and exploration to check for missing values and wrong data entries, and all analyses were conducted using SPSS Version 26 (SPSS Inc, Chicago, IL, USA). The statistical analysis performed in the current study was descriptive (to illustrate the frequency and percentage of demographic and pathological characteristics). Estimating 5-year survival rates was performed using the Kaplan–Meier method; the survival duration was calculated from the date of resection and designating the patient alive or dead as the censor in survival analysis. At the same time, the outcome differences were illustrated through the log-rank test by assigning a *p*-value of <0.05 as the significance limit. The potential prognostic variables were analysed using simple and multivariate logistic regression analysis conducted via the Cox proportional hazards model. A simple Cox regression analysis was used at a univariant level for screening independent variables that are regarded as prognosticating factors with outcomes showing significant *p*-values of <0.25, which were afterwards included in the multivariate analysis to further model the prognosticating factors. Using the Cox Multiple Regression Model CMRM, the stepwise assortment method was employed in variables selection; likewise, the best variable outcomes with a *p*-value < 0.05, whose prognosticating ability was not just significant, but when compared to the other variables in the model, they had been independent, were identified. Additionally, in the preliminary analysis process, the two-way interaction terms amongst the variables were used to check for multicollinearity, proportional hazards assumption, and fitness of the model adequacy, thus finally creating a final model of the factors that had a significant independent connection with survival in the multivariate survival analysis. The final model was illustrated with the adjusted hazard ratio (HR) with a 95% confidence interval (CI) and a significant level of 0.05. Lastly, a t-test of independent variables was used to investigate the association between the prognosis factors (tumour stages and tumour grades) and comorbidity status to show whether this association is significant or due to chance.

## 3. Result

Table 3 illustrates the demographic records. The study involved 114 patients with diagnosed colorectal cancer receiving or being treated at the surgical unit of HUSM. More than two-thirds of the population (89.5%) were Malay, while Indians (5.3%) and Chinese (5.3%) accounted for the remaining races. The most affected age group was 50–69.9 years, estimated at 45.6%. The job status depicted as “occupation” revealed that most patients were already retirees (59.65%). Regarding habitual history, approximately 40.4% of the studied population were smokers, with the male group having the most smokers (95.7%), while 14.0% were ex-smokers and 45.6% were non-smokers (*p* = 0.001).

Regarding tumour location, the left colon (47.4%) was the most recorded tumour site; 33.3% were in the rectum, while the remaining 19% were in the right colon. The staging was illustrated using the Duke’s stage and TNM staging. Duke C (66.6%) was the most observed stage, while Duke A was the least. Similarly, TNM stage-3 (68.4%) was the most recorded stage, while TNM stage-1 (1.8%) was the least reported. In both stagings, the most frequent presentation was Duke C and stage 3 (TNM staging), and the male group was the most concerned; M: F of 60.5% to 39.5% for Duke C and 64.1% to 36% for TNM stage 3. Additionally, regarding tumour grading, as many as 64.9% of the tumours were classified as “moderately graded”, 24.6% were “well-graded”, and about 10.5% were designated as “poorly graded”. The histopathology pattern of CRC illustrated adenocarcinoma as the most expressed morphology (86.0%,) while the remaining morphology showed mucinous adenocarcinoma to be 10.5% and signet-ring adenocarcinoma to be 3.5%. Regarding lymphovascular invasion, 38.6% of the cases were undetected, while 62.9% were positive. Further, 50.9% of CRC patients had metastases in the liver, which was the most affected metastasis site, and there were about 12.3% metastases in the lungs (*p* = 0.011). Regarding the survival status outcomes of the studied patients, death was recorded for 20 patients (17.5%).

In all cases, per rectal bleeding was reported in 43.9% of the population, with the majority being associated with males (68.0%). Regarding the preoperative assessment of carcinoembryonic antigen CEA level among the patients, the majority of CRC patients (63.2%) had >5 ng/mL CEA value (considered abnormal), with males accounting for 72.2% of the CEA value (*p* = 0.032).

Table 4 illustrates the 5-year survival proportion performed using Kaplan–Meier analysis. The parameters represent the demographic and clinicopathological data of the selected patients with confirmed CRC. The analysis outcome showed that those patients who were older than 70 years, who were male, who had metastasis present, who presented with per rectal bleeding and were classified as Duke C and with the tumour in the rectum had the lowest survival rate. From the result, there was a significant difference of median survival time between males (median = 62.1) and females (median = 67.1), *p*-value = 0.026. There was no significant difference of median survival time in the ages 50–69 years old (median = 57.6) and the 70–100 years old (median = 54.1), *p*-value = 0.366. A significant difference in median survival time was noted between those with “presence of metastasis” (median = 32.1) and those with the absence of metastasis (median = 64.5), *p*-value = 0.042. There was no significant difference in median survival time between Duke B (median = 62.0) and Duke C (median = 59.1), *p*-value = 0.941. Still, those classified as Duke B had a higher survival median time than Duke C. There was no significant difference in median survival time between the left site (median = 67.0) and right site (median = 64.0), *p*-value = 0.754. There was no significant difference of median survival time between the left site (median = 67.0) and rectum site (median = 50.0), *p*-value = 0.058. There was no significant difference of median survival time between the right site (median = 64.0) and rectum site (50.0), *p*-value = 0.103. However, those with tumours on the left side had a higher median survival time (67.0), followed by those on the right site (64.0), while those with the rectum site had the lowest median survival time (50.0). The overall median survival time was 62.0.

Table 5 illustrates the analysis of the prognostic factors performed using simple and multiple Cox regression analyses by using the log-rank test to investigate the factors that influence survival and prognosis in CRC. The predictive parameters investigated in this study were gender, age group, ethnicity, occupation status, habitual lifestyle, bleeding per rectum, metastasis presence, metastasis site, tumour location, tumour histopathology, family history, Duke’s stage, tumour grade, lymphovascular invasion, preoperative level of CEA, and modality of treatment. A simple Cox regression analysis was performed on all the potential predictor variables. From the outcome of the simple cox regression analysis, with the significant *p*-values less than 0.25 (*p*-value < 0.25) set in the crude or unadjusted predictive variables for the analysis (simple Cox regression analysis) and prognostic parameters outcomes with a *p*-value < 0.25 being considered as significant clinical importance. Following this selection, multiple Cox regression was employed to further prognosticate this parameter. The multiple Cox proportional hazard regression analysis was performed using the forward and backward LR methods to obtain the adjusted hazard ratio. Only outcomes with a *p*-value of less than 0.05 (*p*-value < 0.05) were retained in the final model.

Table 5 shows the result of a simple Cox proportional hazard regression analysis; those with *p*-values < 0.25 were selected and further examined in the multiple Cox regression analysis (proportional hazards model). From the outcome of the proportional hazards model, five predictor variables had a significant crude hazard ratio (*p*-value < 0.05). For gender, male patients had about a 3.62-fold increased risk of dying from colorectal cancer in an at-risk range of 2–12 than the female patients (*p*-value = 0.040). Regarding the tumour site, the left side was 69% less at risk of dying than the rectum site (*p*-value = 0.046), and the right site was 66% less at risk of dying than the rectum site (*p*-value = 0.101). Those with metastases were 2.7 times more at risk of dying than those with no metastases (*p*-value = 0.010). For the metastasis site, the patients with metastases in the liver were 5.0 times more at risk of dying from CRC than none (*p*-value = 0.031), those with metastases in the lungs were 4.0 times more at risk of dying than none (*p*-value = 0.020), and those with metastases to more than one site in the body, the “others” group, were 3.0 times more at risk of dying than “none” (*p*-value = 0.039). Patients with lymphovascular involvement have a three-fold increased risk of dying in an at-risk range of 2–5 times (95% CI) than patients with no lymphovascular involvement (*p*-value = 0.021). Furthermore, patients with CEA levels greater than 5ng/mL had a two -fold increased risk of dying in an at-risk range of 1–3 times (95% CI) than patients with CEA less than 5ng/mL (*p*-value = 0.001). All the predictor variables with a crude hazard ratio *p*-value less than 0.25 were modelled in the multiple Cox proportional hazard regression, and only “Gender”, “Presence of metastases”, “Metastasis site”, “lymphovascular invasion”, and “CEA level” remained significant in the final model for multiple Cox proportional hazard regression analysis.

Patients’ Charlson Comorbidity Index Score CCIS measurement. CCIS (Table 6) was used to illustrate chronic disease status/burdens and it is regarded as the most employed comorbidity scale by oncologists [17]. It received considerable appraisal for its vast usage in medicine due to its high reliability, usage-friendliness, easy extraction from other indices, and distinctive correlation with health status [18]. From the results of the present study, the majority of the patients had low CCIS between 0–3, indicating that fewer comorbidities were reported by the participating patients. CCIS of 0 (no comorbidity) was reported by 13 patients (24.5%) and 9 patients (14.9%), CCIS of 1 was reported by 99 patients (17.0%) and 6 patients (9.8%), CCIS of 2 was reported by 8 patients (15.1%) and 13 patients (21.3%), and CCIS of 4 was reported by 11 patients (20.8%) and 10 patients (16.4%) for male and female, respectively. Although fewer patients had a high CCIS, meaning more comorbidities, the highest CCIS of 6 was reported by (5.7% and 8.2%) and CCS of 5 was reported by (9.4% and 14.8%) for both male and female, respectively. From the outcome, the male population recorded the highest CCIS and, through the CCIS, the patients can further be categorized into the level of comorbidity severity state.

Table 7 depicts the patients’ Charlson Comorbidity Score-Category Range (N = 114). The participating patients’ level of disease severity status was classified into four groups using the CCIS obtained in Table 4 above. The outcomes show that most of the patients belong to the “None” and “Mild” categories for both male and female group, while about a quarter belongs to the “Severe” category. The first category “Non-group” represents the patients with no history of comorbidities and are reported to be 9 patients (14.8%) and 13 patients (24.5%) for males and females, respectively. In both gender groups, the category with the lowest number was reported in the “Severe category” recording the lowest with 14 patients (23.0%) and 8 patients (15.1%) for the male and female categories, respectively. This group also represents the patients with the most comorbidity burdens. Lastly, the moderate comorbidity in the “Moderate category” was reported to have 19 patients (31.1%) and 15 patients (28.3%) male and female, respectively.

Table 8 below illustrates the relationship between tumour stages and the medical comorbidity status of patients with colorectal cancer. This section describes the associative effect of the tumour stage on medical comorbidities status using a differential statistical tool (independent t-test analysis). Although the result failed to show a significant mean difference in tumour stages among the comorbidity categories, F (4, 83) = 0.92, *p*-value = 0.489. However, the mean stages of patients in the “Severe group”, 44.84 (SD = 5.76), were higher than in the “Non-group” mean, 42.08 (SD = 13.48), “Mild group” mean, 41.08 (SD = 7.39), and “Moderate group” mean, 41.50 (SD = 6.57). This outcome shows that the colorectal cancer patients with more comorbidity numbers and who belong to the severe comorbidity category have the highest mean value and possible late stage of the disease.

Table 9 illustrates the relationship between tumour grades and the medical comorbidity status of patients with colorectal cancer. This section showed a significant mean difference in tumour grades between the medical comorbidity status categories of the patients with colorectal cancer. There was a congruent increase in the mean from the mild to severe comorbid status categories, thus indicating that there is a health correlation of predictive ability of tumour grades with the level of comorbidity status of patients diagnosed with CRC. The mean of patients in “Severe group” 40.52 (SD = 4.90) was higher than in the “Non-group” mean, 33.34 (SD = 9.48), “Mild group” mean, 35.76 (SD = 6.19), and “Moderate group” mean, 38.37(SD = 5.46). This outcome further corroborates the prognostic predictive importance of tumour stages and tumour grades in the diagnosis and prognosis of colorectal cancer as shown that patients with more comorbidity numbers and who belong to the severe comorbidity category have the highest mean value and advanced stages.

## 4. Discussion

This study analysed the influence of colorectal cancer on patients’ life expectancy (survival and prognosis status) via socioeconomic and clinicopathology factors using data on all patients being diagnosed and treated in HUSM. Colorectal cancer (CRC), otherwise known as bowel cancer, colon cancer, or rectal cancer, is the third most common cancer believed by surgeons to have an effective way to reduce the risk factors for cancer if patients are screened for CRC routinely, beginning at age 45 to ensure early diagnosis along with the reduction in the leading risk factors, such as smoking cessation [19]. The percentage of colorectal cancer persons who are still alive after a particular number of years is known as colorectal cancer survival rates. Many colon cancer statistics use a five-year survival rate to estimate the therapy outcome success. However, determining the effectiveness and significance of those treatments on life expectancy can probably take several years due to inconclusive data on the prognosis factors that best predict mortality in CRC [20,21,22]. This study investigated 114 patients diagnosed with CRC who were being treated in HUSM to identify what factors were important in predicting the survival rate. Determining survival factors to estimate the survival rates would ensure a potential benefit in life if cancer-related survival obstacles and differences could be identified and eliminated.

**Figure 2:**
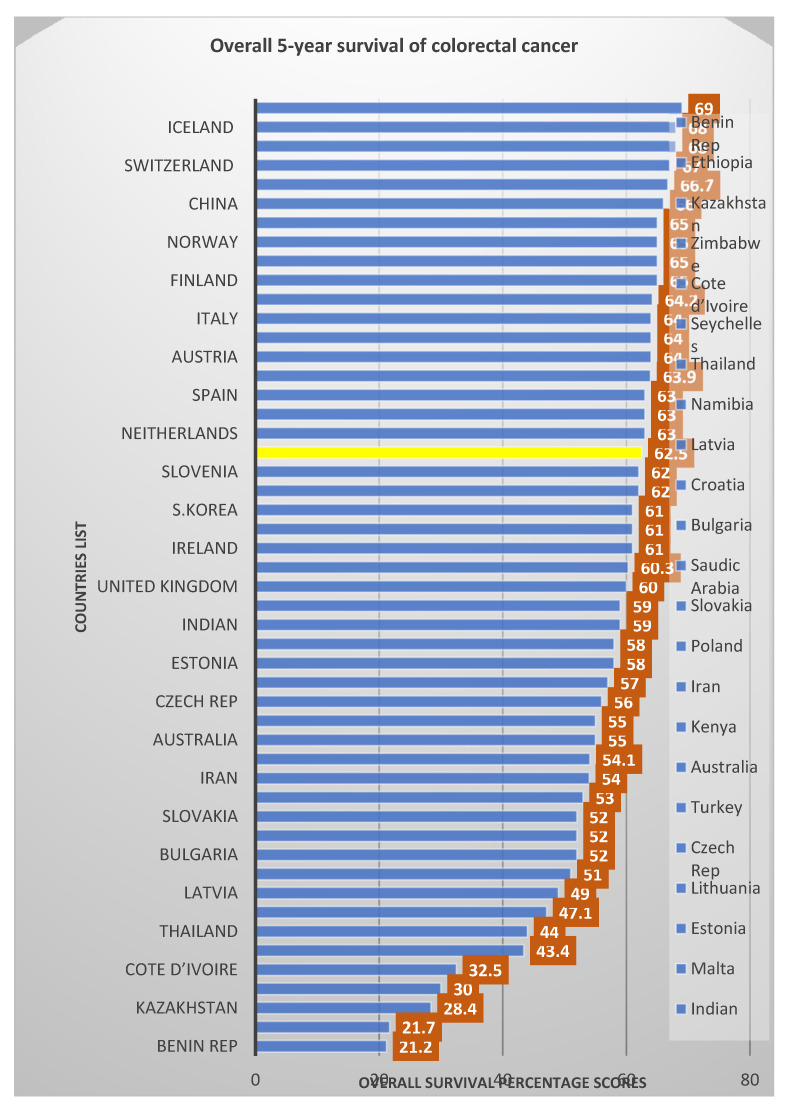
Overall survival percentage score as reported for the different countries in comparison with the present study’s percentage score.

Although individual and geographical survival rates for CRC vary, this research-based study aimed to look at the various prognostic factors that influence the survival rate of CRC patients in HUSM. This study outcome shows that the overall five-year survival rate in patients with colorectal cancer was 62.5%, which is similar to the data (60%) reported by Barnous, Somi [23] and 64.5% reported from the Surveillance, Epidemiology and End Results (SEER) Program from 2008 to 2014, as well as in the United States (64.8%) [24], Australia (63.4%) [25], including the Asian nations of China (60.1%) [26], Japan (68.4%) [27] (68.4%), South Korea (60.0%) [28], and Iran (58.5%) [29]. Given the advanced presentation of CRC in the present study among the age groups, the elderly age group (70 years and above) have the poorest survival rate (54.1%) among all the age groups, just as reported in a study in the UK, which revealed that the incidence rates of CRC in the UK are primarily diagnosed in persons aged 75 and above, the highest among 85–89-year-olds, and this groups is associated with the poorest survival prognosis [30,31]. Among the total population of 114 in this study, 20 (17.5%) patients died, amongst which the majority were male, 16 (80%) compared with 4 (20.0%) of female patients, resulting in a significant higher 5-year survival rate for female patients (67.1%) than 62.0% for male patients (*p*-value = 0.026). Comparing our studies overall 5-years survival rates with rates published from different regions in Figure 2 below, there is a conformity in the trend for survival outcomes among patients diagnosed with CRC. 

Although several studies have tried to identify the many factors that can foretell patient survival after diagnosis, life expectancy has not increased substantially. This is partly because CRC prognosis varies substantially with socioeconomic and histopathological status in countries. From the outcome of the present study, it was revealed that “gender”, “metastasis presence”, “metastasis site”, “Lymphovascular permeation”, and “CEA level” demonstrated the most significant prognosticating power in the multivariant regression analysis. However, “metastasis presence” and “metastasis site” were the most potent prognosticating factors, showing the highest HR. The findings correlate with the results from other studies that some clinicopathological factors, such as tumour site [32], tumour metastasis and metastasis site [33], comorbidity presence and number [34], CEA level [35], and tumour histopathology [36], demonstrate better prognosis in CRC patients. CRC patients with “No metastases” are considered to have a better survival status than those with metastases, 64.5% vs. 32.1%, respectively. Countless patients with distant metastasis present with advanced disease (advanced stage), even with the advancement in medical treatment and diagnostic methods, but at this stage, the treatment is usually palliative and the survival estimate is very low. This study revealed that metastases in the liver showed the most significant prognostic factor, almost five times the risk of death from CRC compared to those without metastases (*p*-value = 0.039). A similar trend was observed for metastases in the lungs; those patients with metastases in the lungs have four times the risk of death compared to those without metastases to another site. A study by Li, Wang [37] revealed that the liver is the most affected metastasis site for patients with CRC, and they exhibited the poorest survival outcome. The anatomical situation of the liver and its portal circulation serves as a vital confluence for metastasis and recurrence routes for advancing tumours such as CRC.

Carcinoembryonic antigen CEA level is an independent factor associated with poorer CRC prognosis and has a higher prognostic value; however, its role in CRC progression remains controversial. A preoperational rise in the serum titre is alarming for abnormal cell growth such as cancer. A rise in the postoperative follow-up critically implies a possible progression or recurrence of the disease because this high value indicates the disease advancement state [38]. CEA is a protein commonly seen in foetal tissues; however, after birth, CEA levels decline to a low level, almost to zero in adults. In cancer, i.e., CRC, the levels rise to higher figures. The CEA test determines the amount of CEA levels in the blood, which in turn serves as a prognostic marker for CRC detection [39,40]. In this study, CEA titre was shown to be enormously significant in both COX regression analyses; patients with CEA levels > 5 ng/mL had almost three times the risk of death compared to those with CEA levels less than 5 ng/mL (*p*-value = 0.001). A study by Wiratkapun, Kraemer [41] reported that a high preoperative CEA value identifies patients with the poorest prognosis, and their study also reported that patients with preoperative serum CEA levels within normal ranges have a significantly higher illness-free survival rate than those with serum CEA levels of ≥5 ng/mL [41]. Although the gender predilection for CRC is controversial and not rubber-stamped [42], according to pre-clinical and clinical research reports, there are sex- and gender-linked changes in CRC development. In CRC, both hereditary and environmental variables are thought to impact sex and gender disparities. While some studies have found that age and sex have little or no bearing on the risk of death in patients with colorectal cancer [43], the present study outcome shows gender to be a significant predictor of death (survival) as the male gender has about a three-fold increased risk of dying from colorectal cancer in an at-risk range of 2–9 times (95% CI) than the female patients (*p*-value = 0.040). Similar findings have also been reported in several studies [44,45,46,47,48,49].

In this study, the prognosis was not shown to be affected by demographic factors, such as age, ethnicity, chronic status (smoking habit), and occupation. This was also reported by [40,50]. Surprisingly, other potentially significant factors, such as Duke’s stage, tumour stage, tumour grading, tumour location, and tumour histology, which were believed to influence the relationships between response to treatment and survival time of CRC, were not shown by the present study as decisive prognostic factors, and similar results were reported by [51,52]. This was contrary to some pre-existing findings that uphold this former fact [30,45,53]; they revealed that, regarding tumour location, patients with CRC located in the colon have a better survival estimate than those with cancer located in the rectum, while CRC patients with an advanced pathological tumour stage, such as stage 4 or Duke D, or poor tumour grade have poor survival outcomes. Although some studies have found tumour stage (Duke’s or TNM classes) crucial in predicting survival outcomes [54,55], it is still considered inconclusive probably because many CRC patients usually present at the late stage of the disease when the severity has become intense with a less success rate of treatment outcome. Instead, several studies reported these factors: tumour staging and location as potentially significant predictors of treatment outcome but not definitive prognosis factors [56,57,58]. The latter studies reported a reverse trend, indicating that tumour stage and grade have been lowered in most outcomes, pointing to the uncertain predictive potential of tumour stage and grade. Lee, Park [57] reported that higher clinical T and N stages were not predictive factors for pathologic CRC in the multivariate analysis. The present study could not portray a tumour site as a significant predictor of survival status, even though [44] reported a tumour in the rectum to be a significant predictor of survival status in their multivariable technique. In lieu of this outcome, the severity of the tumour should be based on summative information on the histopathological and clinical presentation before a comprehensive treatment plan can be administered to the patient. Nonetheless, gender, lymphovascular status, metastasis presence, and site of metastasis play a significant role in predicting the chances and rate of survival in patients with CRC. In this study, the multivariate analyses presented in this paper focused on the interdependence of the prognosticating factors based on their impact on the endpoints of interest, thus revealing the independent predictive capability of these factors (e.g., metastasis presence and site of metastasis). Additionally, it should be mentioned that, while some factors such as smoking and being poorly graded were revealed to be significant only in the univariate analyses, their lack of independent predictive capability in the multivariant analyses in the CRC cases was demonstrated.

One common phenomenon is that male patients are at the highest risk of CRC diagnosis, especially among the elderly [59], and, in this study outcome, the proportion of male patients associated with CRC is about two-fold greater than their female counterparts M: F: 64.9% and 35.1%, respectively. Although, the reason could be multi-factorial, ranging from biological and behavioural factors to high red and processed meat dietary intake, high alcohol intake, chronic smoking history, and considerable proclivity for visceral fat deposit for males, all of which are considered high-risk factors for CRC [30,45,53,60,61,62,63,64]. With parameters such as tumour grades and tumour stages regarded as some of the most reliable tools for CRC prognosis and treatment success, chronic diseases or state of health (comorbidity status), which are presumed different or can co-exist with the principal diagnosis of interest (CRC), can mask spot diagnosis and hinder treatment outcome. For this major reason, it becomes mandatory to identify and classify the comorbidity burden of the patient for the clinicians to decide how vigorously to treat such patients, especially patients with a high Charlson Comorbidity Index Score (CCIS). Thus, it a necessity to have a standardised and accurate tool (Charlson Comorbidity Index Scale) for determining comorbidity burdens. The study showed a statistically significantly increasing mean difference between the tumour grades and comorbidity status, as the mean comorbidity status of patients in the “Severe group”, 40.52 (SD = 4.90), was higher than that in the “None group” (mean = 33.34, SD = 9.48) and “Mild group” (mean = 35.76, SD = 6.19), *p*-value 0.05. However, the study failed to show a statistically significant mean difference between the tumour stages and comorbidity status; the mean comorbidity status of patients in the “Severe group”, 44.84 (SD = 5.76), was higher than that in the "Non-group" (mean = 42.08, SD = 13.48) and “Mild group” (mean = 41.19, SD = 7.29), *p*-value = 0.489. This outcome corroborates several studies report that CCI is a reliable prognosticator of mortality rate in chronic diseases, such as kidney cancer [65], head and neck cancer [66], and arthritis [67]. 

Loss in life expectancies is a valid metric for determining the effects of a cancer diagnosis over the remainder of one’s life. It offers absolute values of very high certainty on which both cancer and other causes of death can be evaluated. Even though this study showed that loss in life expectancy measures (prognosis factors) provides valuable summaries of cancer outcomes, this study was not without some limitations because no direct comparisons with some other prognosis measures were examined. Ab initio, more research will be needed to explain these disparities between prognosis systems in advanced cancer patients. Despite these limitations, crucial factors that can predict and influence the prognostic outcome of CRC patients were categorically outlined.

## 5. Conclusions

CRC is one of those cancers that can be mostly avoided if detected early because, when CRC is detected while it is still localised, survival is considerably high. Though several studies have tried to identify factors that may foretell patient survival after a colorectal cancer diagnosis, life expectancy has not significantly increased. The result from this present study supports the several already released reports that the histopathological parameters (gender, metastasis presence, site of metastasis, CEA level, and lymphovascular permeation) of patients may be the best factors for predicting prognosis and survival in colorectal cancer patients, while CCI provides a comprehensive disease burden of the patients. However, factors such as habitual lifestyle (smoking habit), tumour grade (poorly graded), and comorbidity number that presented significant outcomes at the univariant level should also be considered. From the study’s outcome, early screening strategies will ensure CRC early detection and promote better survival outcomes.

## 6. Merits and Recommendations of the Study

The findings released in this study will benefit the design and analysis of colorectal cancer’s impact on survival for future studies. This study showed that future treatment protocols for advanced-stage colorectal cancer should take note of the heterogeneity of the affected patients concerning the importance of the prognosticating markers by judiciously examining the histopathology presentations of the disease.

## Figures and Tables

**Table 1:** Summary of lists of Charlson Comorbidity Diseases.

Comorbid/Diseases	Assigned Weight Index	Comorbid/Diseases	Assigned Weight Index
1. Congestive heart failure	1	10. Hemiplegia/ Paraplegia	1
2. Peripheral vascular disease	1	11. Dementia	1
3. Chronic pulmonary obstructive disease	1	12. Moderate or severe renal disease	2
4. Myocardial infarction	1	13. Diabetes with end organ damage	2
5. Rheumatologic disease	1	14. Moderate–severe liver disease	2
6. Cerebrovascular disease	1	15. Any tumour, including leukaemia and lymphoma	2
7. Peptic ulcer disease	1	16. Metastatic solid tumour	6
8. Mild liver disease	1	17. AIDS	6
9. Diabetes (uncomplicated)	1		

**Table 2:** Summary of Charlson Comorbidity Score-Category Range (CCSCR).

Charlson Comorbidity Score-Category Range (CCSCR)	Charlson Comorbidity Score (CCS)
None	0
Mild	1 or 2
Moderate	3 or 4
Severe	≥5

**Table 3:** Demographic data of the patients.

		Male	Female				Male	Female	
Characteristics	Total ^a^	N (%)	N (%)	*p*-Value	Characteristics	Total	N (%)	N (%)	*p*-Value
Overall	N =114	74 (64.9)	40 (35.1)		Overall	N = 114	74 (64.9)	40 (35.1)	
Age group 16–49 50–69.9 70–100	26 (22.8) 52 (45.6) 36 (31.6)	18 (69.2) 36 (69.2) 20 (55.6)	8 (30.8) 16 (30.8) 16 (44.4)	0.364 ^b^	Metastases Yes No	58 (50.9) 56 (49.1)	46 (79.3) 28 (50.0)	12 (20.7) 28 (50.0)	0.001 *^b^
Ethnicity Malay Chinese Indian	102 (89.5) 6 (5.3) 6 (5.3)	66 (64.7) 4 (66.7) 4 (66.7)	36 (35.3) 2 (33.3) 2 (33.3)	0.991 ^b^	Metastasis site Liver Lungs Others None	36 (31.6) 14 (12.3) 8 (7.0) 56 (49.1)	28 (77.8) 12 (85.7) 6 (75.0) 28 (50.0)	8 (22.2) 2 (14.3) 2 (25.0) 28 (50.0)	0.011 *^b^
Occupation Working Retiree	46 (40.4) 68 (59.6)	34 (73.9) 40 (58.8)	12 (26.1) 28 (41.2)	0.098 ^b^	Per rectal bleeding Yes No	50 (43.9) 64 (56.1)	34 (68.0) 40 (62.5)	16 (32.0) 24 (37.5)	0.541 ^b^
Habitual Smoking Ex-smoker Non-smoker	46 (40.4) 16 (14.0) 52 (45.6)	44 (95.7) 10 (62.5) 20 (38.5)	2 (4.3) 6 (37.5) 32 (61.5)	0.001 *^b^	CEA level ≤ 5 > 5	42 (36.8) 72 (63.2)	20 (47.6) 52 (72.2)	22 (52.4) 20 (27.8)	0.032 *^b^
Tumour site Left Right Rectum	54 (47.4) 22 (19.3) 38 (33.3)	20 (37.0) 16 (72.7) 38 (100)	34 (63.0) 6 (27.3) 0 (0)	0.001 *^b^	Treatment modality Surgery only Surgery + Chemo/Radio Surgery + Chemo + Radio Chemo or Radio	32 (28.1) 62 (54.4) 14 (12.3) 6 (5.3)	20 (62.5) 40 (64.5) 12 (85.7) 2 (33.3)	12 (37.5) 22 (35.5) 2 (14.3) 4 (66.7)	0.046*^b^
Duke staging Duke A Duke B Duke C Duke D	6 (5.3) 22 (19.3) 76 (66.7) 10 (8.8)	4 (66.7) 16 (72.7) 46 (60.5) 8 (80.00	2 (33.3) 6 (27.3) 30 (39.5) 2 (20.0)	0.524 ^b^	Comorbidity HTN DM Others None	24 (21.1) 12 (10.5) 48 (42.1) 30 (26.3)	14 (58.3) 10 (83.3) 26 (54.2) 24 (80.0)	10 (41.7) 2 (16.7) 22 (45.8) 6 (20.0)	0.053 *^b^
TNM stage Stage 1 Stage 2 Stage 3 Stage 4	2 (1.8) 22 (19.3) 78 (68.4) 12 (10.5)	2 (100.0) 12 (54.5) 50 (64.1) 10 (83.3)	0 (0) 10 (45.5) 28 (35.9) 2 (16.7)	0.269 ^b^	Comorbidity Number Single ≥2 None	40 (35.1) 44 (38.6) 30 (26.3)	28 (70.0) 22 (50.0) 24 (80.0)	12 (30.0) 22 (50.0) 6 (20.0)	0.021 *^b^
Tumour grading Well Moderately Poorly	28 (24.6) 74 (64.9) 12 (10.5)	22 (78.6) 42 (56.8) 10 (83.3)	6 (21.4) 32 (43.2) 2 (16.7)	0.044 *^b^	Family history Malignancy Other malignancy Adenomatous polyposis None	28 (24.6) 20 (17.5) 4 (3.5) 62 (54.4)	18 (64.3) 12 (60.0) 4 (100) 40 (64.5)	10 (35.7) 8 (40.0) 0 (0) 22 (35.5)	0.497 ^b^
Survival status Dead Alive	20 (17.5) 94 (82.5)	16 (80.0) 56 (59.6)	4 (20.0) 38 (40.4)	0.172 ^b^	Clinical features Abdominal Pain/cramp Abdominal Bloating/ distention Weight loss Loss of appetite/ anorexia Anaemia/Pale Tenesmus/constipation/ diarrhoea Bleeding per rectum Nausea/Vomiting/Fatigue Shortness of breath	34 (9.4) 30 (8.3) 52 (14.4) 56 (8.3) 4 (1.1) 64 (17.8) 48 (13.3) 60 (16.7) 12 (3.3)	20 (58.8) 16 (53.3) 15 (28.8) 22 (39.3) 1 (25) 40 (62.5) 36 (75) 22 (36.7) 5 (41.7)	14 (41.2) 14 (46.7) 37 (71.2) 24 (42.9) 3 (75) 24 (37.5) 22 (25) 38 (63.3) 7 (58.3)	0.043 *^b^
Tumour histology Adenocarcinoma Mucinous adenocarcinoma Signet-cell adenocarcinoma	98 (86.0) 12 (10.5) 4 (3.5)	58 (59.2) 12 (100) 4 (100)	40 (40.8) 0 (0) 0 (0)	0.007 *^b^					
Lymphovascular Invasion Present Absent	70 (61.4) 44 (38.6)	44 (62.9) 30 (68.2)	26 (37.1) 14 (31.8)	0.562 ^b^					

* Denotes significant *p*-value; a: column underneath “Total” is descriptive; b: chi-squared test.

**Table 4:** The 5-year survival proportion performed using Kaplan–Meier analysis.

Variables	Survival Rate (%)	95% Confidence Interval (CI)		*p*-Value
		Lower Limit	Upper Limit	
	Overall (62.5%)			
Gender Male Female	62.1 67.0	48.992 59.519	58.129 68.581	0.026 *
Age group 50–69.9 70–100	57.6 54.1	51.942 47.541	63.224 60.590	0.366
Metastases status Present Absent	32.1 64.5	22.963 50.312	54.628 81.143	0.042 *
Per rectal bleeding No Yes	52.7 42.8	28.9 29.5	73.631 59.391	0.041 *
Duke staging Duke B Duke C	62.0 59.1	46.742 44.572	72.258 63.560	0.914
Tumour site Left Right Rectum	67.0 64.0 50.0	54.950 52.253 45.696	65.563 67.211 55.721	- 0.754 0.058

* Denotes significant *p*-value.

**Table 5:** Prognostic factors using simple and multiple Cox proportional hazards regression models.

Factors	CHR (95% CI)	Wald	*p*-Value *	Factors	CHR (95% CI)	Wald	*p*-Value	AHR (95% CI)	*p*-Value *^†^
Age group 16–49 50–69.9 70–100	1 0.66 (0.25, 1.74) 0.28 (0.06, 1.25)	2.78 0.69	0.095 0.405	Gender Female Male	1 4.68 (2.06, 12.81)	4.20	0.020	1 2.62 (1.56, 9.81)	0.040 *^†^
Ethnicity Indian Malay Chinese	1 0.28 (0.03, 2.25) 0.00 (-)	1.44 0.00	0.231 0.981	Metastases No Yes	1 4.60 (2.18, 10.94)	5.10	0.024	1 3.76 (1.89, 7.32)	0.010 *^†^
Occupation Retiree Working	1 0.52 (0.20, 1.36)	1.79	0.181	Lymphovascular Invasion Absent Present	1 3.09 (1.44, 8.75)	0.04	0.043	1 2.94 (1.99, 5.92)	0.021 *^†^
Habitual Non-smoker Smoking Ex-smoker	1 1.90 (0.53, 4.30) 2.13 (0.68, 6.73)	0.58 1.67	0.048 0.096	CEA level ≤ 5 > 5	1 3.25 (1.99, 7.20)	0.22	0.002	1 2.43 (1.49, 5.80)	0.001 *^†^
Tumour grading Well Moderately Poorly	1 1.04 (0.29, 3.70) 1.90 (0.23, 6.35)	0.04 0.01	0.835 0.047	Tumour site Rectum Left Right	1 0.31 (0.10, 0.98) 0.34 (0.09, 1.24)	3.99 2.69	0.046 0.101	-	-
Duke staging Duke A Duke B Duke C Duke D	1 1.44 (0.26, 8.01) 0.84 (0.19, 3.80) 1.78 (0.25, 12.86)	0.32 0.17 0.05	0.680 0.818 0.570	Metastasis site None Others Lungs Liver	1 3.04 (1.93, 9.90) 4.94 (1.98, 20.36) 5.42 (1.03, 33.99)	3.39 4.88 5..98	0.010 0.020 0.046	1 3.11 (1.46, 7.51) 4.42 (1.20, 12.36) 5.04 (1.71, 19.05)	0.031 *^†^ 0.020 *^†^ 0.039 *^†^
TNM stage Stage 1 Stage 2 Stage 3 Stage 4	1 0.99 (0.18, 5.54) 1.05 (0.24, 4.70) 1.06 (0.09, 11.94)	0.00 0.01 0.00	0.999 0.949 0.966	Family history None Malignancy Other malignancy Adenomatous polyposis	1 1.21 (0.48, 3.09) 0.00 (-) 3.04 (0.64, 14.40)	0.16 0.00 1.97	0.686 0.979 0.161	-	-
Comorbidity Type None HTN DM Others	1 1.97 (0.55, 7.02) 0.00 (-) 1.02 (0.31, 3.35)	1.10 0.00 0.00	0.295 0.988 0.981	Treatment modality Chemo or Radio Surgery only Surgery + Chemo/Radio Surgery + Chemo + Radio	1 0.53 (0.06, 4.66) 0.50 (0.06, 4.17) 1.67 (0.19, 14.60)	0.33 0.41 0.21	0.568 0.520 0.645	-	-
Comorbidity N None Single ≥2	1 1.17 (0.33, 4.18) 1.23 (0.38, 3.98)		0.809 0.035	Tumour Histopathology Unidentified Adenocarcinoma Mucinous adenocarcinoma Signet adenocarcinoma	1 0.17 (1.95, 8.18) 0.23 (2.38, 10.98) 0.93 (2.38, 7.98)	0.83 0.72 0.41	0.12 0.411 0.321	-	-
Per rectal bleeding No Yes	1 1.06 (0.42, 2.68)	0.02	0.901						

* Denotes significant *p*-value; **^†^** *p* for Wald statistic, HR = hazard ratio; CI = confidence interval; CEA = carcinoembryonic antigen.

**Table 6:** Patients’ Charlson Comorbidity Index Score CCIS measurement (N = 114).

Variables	Male n (%)	Female n (%)	ᵡ (df)	*p*-Value
Charlson Comorbidity Index Score CCIS			8.6 (7)	0.373
0	9 (14.9)	13 (24.5)		
1	6 (9.8)	9 (17.0)		
2	13 (21.3)	8 (15.1)		
3	9 (14.8)	4 (7.5)		
4	10 (16.4)	11 (20.8)		
5	9 (14.8)	5 (9.4)		
6	5 (8.2)	3 (5.7)		

**Table 7:** Patients’ Charlson Comorbidity Score-Category Range (N = 114).

Variables	Male n (%)	Female n (%)	ᵡ (df)	*p*-Value
Charlson Comorbidity Score-Category Range (CCSCR)			3.6 (3)	0.434
None (CCS: 0)	9 (14.8)	13 (24.5)		
Mild (CCS: 1–2)	19 (31.1)	17 (32.1)		
Moderate (3–4)	19 (31.1)	15 (28.3)		
Severe (CCS:>5)	14 (23.0)	8 (15.1)		

**Table 8:** Independent *t*-test to determine the association between tumour stage and medical comorbidity status among patients with colorectal cancer.

Charlson Comorbidity Score-Category Range (CCSCR)	Mean (SD)	F (df)	*p*-Value
None	42.08 (13.48)	0.92 (4.83)	0.489
Mild	41.19 (7.29)		
Moderate	41.50 (6.57)		
Severe	44.84 (5.76)		

**Table 9:** Independent *t*-test to determine the association between tumour grades and medical comorbidity status of patients with colorectal cancer.

Charlson Comorbidity Score-Category Range (CCSCR)	Mean (SD)	F (df)	*p*-Value
None	33.34 (9.48)	0.74 (3.63)	0.050
Mild	35.76 (6.19)		
Moderate	38.37 (5.46)		
Severe	40.52 (4.90)		
